# Could high DNA stainability (HDS) be a valuable indicator of sperm nuclear integrity?

**DOI:** 10.1186/s12610-020-00110-8

**Published:** 2020-08-13

**Authors:** Z. Mohammadi, M. Tavalaee, P. Gharagozloo, J. R. Drevet, M. H. Nasr-Esfahani

**Affiliations:** 1 Department of Reproductive Biotechnology, Reproductive Biomedicine Research Center, Royan Institute for Biotechnology, ACECR, Isfahan, Iran; 2 CellOxess LLC, 830 Bear Tavern Road, Ewing, NJ 08628 USA; 3 GReD Institute, Faculty of Medicine, INSERM-CNRS-Université Clermont Auvergne, Clermont-Ferrand, France; 4 Isfahan Fertility and Infertility Center, Isfahan, Iran

**Keywords:** Human spermatozoa, Sperm nuclear integrity, Sperm DNA fragmentation, Sperm DNA condensation, Acridine orange staining, High DNA stainability (HDS), Spermatozoïdes humains, Intégrité nucléaire des spermatozoïdes, Fragmentation de l’ADN des spermatozoïdes, Condensation de l’ADN des spermatozoïdes, Coloration à l’acridine orange, Haute sensibilité à la coloration

## Abstract

**Background:**

The Sperm Chromatin Structure Assay (SCSA®), in addition to identifying the DNA Fragmentation Index (DFI) also identifies High DNA satiability (HDS), supposed to reflect the nuclear compaction of spermatozoa. However, data on what exactly this parameter reveals, its relevance and usefulness are contradictory. In order to shed light on this situation, spermatozoa of a cohort (*N* = 397) of infertile men were subjected to the SCSA®, TUNEL (terminal deoxynucleotidyl transferase-mediated deoxyuridine triphosphate-biotin nick end labeling) and CMA3 (Chromomycin A3) tests. In a smaller subcohort (*N* = 100), aniline blue (AB) and toluidine blue (TB) staining were performed in addition. The objective of this study was thus to answer the question of whether HDS is a relevant and reliable parameter to be taken into account?

**Results:**

HDS does not appear to be a reliable indicator of nuclear immaturity because it shows a weak correlation with the CMA3, AB and TB stains. The low correlation of HDS with sperm DNA fragmentation (TUNEL and SCSA®) and DNA condensation (CMA3, AB and TB) tests suggests that these two parameters could be decoupled. Unlike DFI and TUNEL, HDS has not been shown to correlate with classic clinical situations of male infertility (asthenozoospermia, teratozoospermia or astheno-teratozoospermia).

**Conclusion:**

HDS correlates poorly with most tests that focus specifically on the level of maturity of the sperm nucleus. To our knowledge, this study is the first to compare SCSA®, TUNEL, AB, TB and CMA3 assays on identical samples. It shows the potency, consistency and limitations of each test and the care that must be taken in their interpretation.

## Résumé

**Contexte:**

Le test SCSA® (Sperm Chromatin Structure Assay), en plus d’identifier l’indice de fragmentation de l’ADN (DFI = DNA fragmentation Index), identifie également la susceptibilté à la coloration à l’acridine orange de l’ADN (HDS: High DNA Stainability), censée refléter la compaction nucléaire des spermatozoïdes. Cependant, les données sur ce que révèle exactement ce paramètre, sa pertinence et son utilité sont contradictoires. Afin de faire la lumière sur cette situation, les spermatozoïdes d’une cohorte (*N* = 397) d’hommes stériles ont été soumis aux tests SCSA®, TUNEL et CMA3. Dans une sous-cohorte plus petite (*N* = 100), la coloration au bleu d’aniline (AB) et au bleu de toluidine (TB) a été effectuée en plus. L’objectif de cette étude était donc de répondre à la question de savoir si le HDS est. un paramètre pertinent et fiable à prendre en compte?

**Résultats:**

Le HDS ne semble pas être un indicateur fiable de l’intégrité nucléaire car il montre une faible corrélation avec les tests CMA3, AB et TB. La faible corrélation du HDS avec les tests de fragmentation de l’ADN du sperme (TUNEL et SCSA®) et de condensation de l’ADN (CMA3, AB et TB) suggère que ces deux paramètres pourraient être découplés. Contrairement au DFI et au TUNEL, il n’a pas été démontré que le HDS est. corrélé avec les situations cliniques classiques de l’infertilité masculine (asthénozoospermie, tératozoospermie ou asthéno-tératozoospermie).

**Conclusion:**

Le HDS présente une faible corrélation avec la plupart des tests qui se concentrent spécifiquement sur le niveau de maturité du noyau du sperme. À notre connaissance, cette étude est. la première à comparer les tests SCSA®, TUNEL, AB, TB et CMA3 sur des échantillons identiques. Elle montre la puissance, la cohérence et les limites de chaque test et le soin qui doit être apporté à leur interprétation.

## Introduction

Optimal nuclear sperm condensation is one of the major issues in the male germ cell differentiation program and, to achieve this, mammalian sperm go through a complex process during spermiogenesis and post-testicular maturation (for recent reviews, see: [1–3]). The main objective of extreme cyto-differentiation of sperm is to confer special hydrodynamic properties to the smallest and mobile mammalian cell. At the same time, nuclear compaction protects the paternal genetic material from damage, a necessity for a “silent” cell lacking cytosolic protective activities and unable to develop genetically mediated stress responses and repair itself [4].

Since the advent of assisted reproductive technologies some 25 years ago, the means by which the fertility of the male partner is evaluated is rather an expeditious case. Considering the advances in this filed, the methods or recommendations for semen analysis are still limited to sperm count, motility and morphology monitoring (WHO, 2010) [5]. However, the increased worldwide use of the most invasive ART procedure (intracytoplasmic sperm injection = ICSI) has rendered these evaluations non-essentials in terms of reproductive success. In this context, it appears that in order to improve our understanding of the etiology of male infertility, further and deeper testing is needed. Over the last decade, it has become increasingly clear that an important criterion for reproductive success is the quality and the integrity of the paternal nucleus. There is ample evidence that sperm with nuclear alterations are associated with reproductive failure [6]. Specifically, sperm nucleus fragmentation has been shown to be associated with fertilization failures, delayed embryo development, implantation failures, embryo loss, increased perinatal mortality and an increased incidence of pathologies in offspring ranging from the development of childhood tumors to the development of complex, multifactorial pathologies such as type 2 diabetes, neuro-muscular degenerative syndromes and autistic disorders [7–12].

In addition to DNA fragmentation, the loss of sperm nuclear integrity has multiple faces that can be cumulative, including aneuploidy, single or double-stranded DNA breaks, the presence of abasic sites, abnormal cross-linking of nuclear proteins, nuclear decondensation due to aberrant nuclear protein content or reduced disulfide bridges between protamine rings [13]. The loss of nuclear integrity of sperm can be even more subtle and may involve changes in the epigenetic information they carry, ranging from DNA (e.g. methylation/hydroxymethylation status), to nuclear proteins with the wide range of persistent post-translational histone modifications, to nucleus-associated non-coding RNAs [14, 15]. In this context, it soon became apparent that there was a clinical interest in diagnosing the level of integrity of the paternal nucleus which could be considered an important indicator of reproductive success. Several tests directly or indirectly assessing the level of fragmentation/condensation of the sperm nucleus are available. However, although there is near consensus that an altered paternal nucleus is conducive to reproductive failure [16, 17], there is still no consensus in the clinical community regarding the test of choice for assessing sperm nuclear integrity [18–22]. Conflicting reports have caused much confusion, which explains why sperm DNA integrity tests are still not part of the routine worldwide evaluation of men in infertile situations. Of the many tests available, the one(s) that is(are) the most relevant and predictive of reproductive success has not yet been agreed upon. This is not surprising as the different tests available do not address the same questions and are chemically distinct. Basically, these tests meet two different but related criteria, namely fragmentation of sperm DNA and condensation of sperm DNA. Among the tests available, the acidic aniline blue (AB) stain test targets histones [23–25], while chromomycin A3 (CMA3) competes with protamine to interact with DNA [26]. Toluidine blue (TB) has an affinity for accessible DNA phosphate groups, reflecting poor chromatin organization [27]. The TUNEL (Terminal deoxynucleotidyl transferase mediated dUTP nick-end labelling) assay uses terminal transferase (TdT) to detect the free 3′-OH ends of fragmented DNA [28, 29]. The Comet assay assesses DNA fragmentation based on electrophoretic characteristics [30]. The Sperm Chromatin Structure Assay (SCSA**®**) uses acridine orange (AO: a cell-permeable nucleic acid binding dye that fluoresces green when bound to double-stranded DNA [dsDNA] and red when bound to single-stranded DNA [ssDNA]) to stain sperm DNA that may be denatured by acid treatment or temperature [31–33]. The use of a low pH in the SCSA**®** assay opens the DNA strand at break sites, allowing the AO to access the single-stranded DNA. Specially designed software [31] transforms the flow cytometer data into a DNA Fragmentation Index (DFI) for which thresholds have been determined. The currently established clinical threshold is 25% DFI, above which a man is considered to encounter reproductive problems [31] and may be advised to use ART (mainly ICSI or even TESE/ICSI when the DFI reaches higher values).

Among the tests used to assess the level of sperm nucleus fragmentation, the TUNEL and the SCSA**®** are the most commonly used. Although the TUNEL assay directly assesses DNA breaks, it tends to underestimate the actual level of DNA fragmentation because of its inability to recognize breaks that do not leave 3′-OH ends free, as is the case when breaks are induced by acute oxidative stress [34, 35]. Since its development in the early eighties [36], the SCSA**®** has proven to be by far the most widely used and accepted test for assessing the integrity of nuclear material in semen. Because of its wide use, it is supported by a large amount of data, making it the most robust test for predicting reproductive success [37–44], especially with IUI (intrauterine insemination) and IVF (In Vitro fertilization), while it is not so conclusive with ICSI [45]. It is well accepted in the community that when a person’s DFI is greater than 30%, the probability of pregnancy success is close to zero (for a review, see: [45]). In addition to DFI, another parameter, the percentage of sperm with high DNA stainability (HDS), is provided by the SCSA**®** flow cytometer-assisted assessment. HDS has been hypothesized to reflect the immaturity of the sperm nucleus, which has been proposed to be due to a sub-optimal histone to protamine ratio that affects sperm nucleus compaction and therefore makes it susceptible to DNA damage [32, 33, 46].

Although this hypothesis is theoretically understandable, to date there are no reports in which HDS and histone retention (or protamine deficiency) have been co-controlled. There are, however, reports in which DFI has been monitored in conjunction with protamine assessment (see for example: [47]). To fill this gap, we decided to analyze the relationship between HDS, protamine content and nuclear condensation, as determined by chromomycin A3 (CMA3), aniline blue (AB) and toluidine blue (TB) staining [48]. Since low sperm nuclear condensation due to suboptimal protamine content makes sperm more susceptible to DNA damage, we also tested sperm DNA integrity in the same samples by the TUNEL assay and the SCSA**®**, which allowed us to evaluate in parallel the correlation between these classical sperm DNA fragmentation tests and the CMA3, AB and TB tests.

## Materials & methods

This study was carried out at the Isfahan Fertility and Infertility Center and was approved by the Royan Institute’s ethics committee (IR.ACECR.ROYAN.REC.1398.258). All male patients (*n* = 397, mean age = 36.78 years) who participated in the study signed a consent form.

Semen samples were obtained from each participant by masturbation within a 3–5 day abstinence window. Semen samples were delivered to the laboratory within 10 min of ejaculation. For semen analysis, volume, liquefaction and viscosity were assessed within 15–30 min of ejaculation. All other analyses were started immediately after the evaluation of liquefaction and viscosity. To evaluate the sperm concentration, a counting chamber (Sperm Meter, Sperm Processor, Aurangabad, India) using a LABOMED CxL optical microscope (magnification: 20X) was used and, if necessary, the sperm was diluted (1:10) in 1% formalin in a sodium bicarbonate solution. At least 200 spermatozoa were counted for each sample and the result was expressed in millions per millilitre.

For sperm motility, the semen samples were heated to 37 °C. Sperm motility was assessed by computer-assisted sperm analysis (CASA) using a LABOMED CxL light microscope. Ten μl of semen were loaded into a preheated sperm counting chamber with a cover slide allowing a 20 μm chamber height. At least 200 spermatozoa in at least five fields were evaluated. Four types of sperm movement were defined for each sample (fast progressive, slow progressive, non-progressive and immotile), and the results were expressed as a percentage of “total sperm motility” and “progressive sperm motility”.

Sperm morphology was evaluated according to strict Tygerberg criteria with a trained technician. Papanicolaou staining was used to assess sperm morphology. For each sample, two smears were prepared and 200 sperm were counted. Abnormalities of the sperm head, neck and tail were evaluated at high magnification (× 1000) using a high-resolution (100×) oil-immersion objective and bright-field microscope optics. The results of sperm morphology staining were expressed as “percentage of abnormal sperm morphology” [5].

In addition to conventional analysis of semen samples, the integrity of the sperm nucleus was studied by the SCSA®, TUNEL and CMA3 tests (Experiment I). In 100 of these 397 patients, in addition to the 3 tests mentioned above, the chromatin status of the sperm (condensation level) was also evaluated by staining with aniline blue (AB) and toluidine blue (TB) (Experiment II).

### Evaluation of DNA fragmentation by dUTP terminal deoxynucleotidyl transferase nick-end labelling (TUNEL)

The TUNEL analysis was performed according to the manufacturer’s instructions (Promega, Mannheim, Germany). Briefly, the samples were rinsed with phosphate buffered saline (PBS 1X; pH 7.4) and resuspended at a concentration of 1.10^6^ sperm cells/ml. The samples were then fixed in 4% paraformaldehyde for 30 min and impregnated for 5 min in 0.2% Triton X-100 (Merck, Darmstadt, Germany). The samples were then washed to remove the permeabilising agent. The samples were evaluated with a FACS-Calibur flow cytometer (BD Biosciences, San Jose, CA, USA) and at least 10,000 sperm were counted. The results were reported as % DNA fragmented cells.

### Assessment of DNA fragmentation by the sperm chromatin structure assay (SCSA®)

The SCSA® was conducted in accordance with the recommendations of its developer [36]. After evaluation of the sperm concentration, 2. 10^6^ sperm cells were suspended in TNE buffer (50 mM Tris HCl pH 7.4, 100 mM NaCl, 0.1 mM EDTA; Merck, Darmstadt, Germany) to a final volume of 1 ml. The SCSA was performed on 1/5 aliquot of the spermatozoa suspension to which 400 μl of acid-detergent solution was added. Then 1.2 ml acridine orange (AO) staining solution (Sigma, St. Louis, USA) was added for 30 s. The samples were analyzed with a FAX-Calibur cytometer (BD Biosciences, San Jose, CA, USA). At least 10,000 sperm cells were counted and the results were presented in the conventional manner with the DFI (DNA Fragmentation Index %) and HDS (High DNA Stainability %) scores.

### Assessment of sperm protamine deficiency by chromomycin A3 (CMA3) staining

In short, for each sample, two smears of washed spermatozoa fixed with Carnoy were taken. For staining, 200 μl CMA3 solution (0.25 mg/ml) was added to the smears. The slides were then rinsed 3 times with PBS 1X. At least 200 spermatozoa were evaluated using an epifluorescence microscope (Olympus, Japan) equipped with appropriate filters (460-470 nm) at × 100 magnification. Spermatozoa with low or insufficient protamine content appear light yellow, while spermatozoa with normal protamine content appear dark yellow [46].

### Evaluation of spermatozoa histone content by aniline blue staining

In short, for each sample, two washed spermatozoa smears were taken. The slides were fixed with 3% glutaraldehyde and stained with 5% aqueous aniline blue (AB) in 4% acetic acid. The slides were then dehydrated in successive ethanol baths (70, 96 and 100%) and exposed to xylol for 5 min. The slides were then covered with Entelane. For each sample, at least 200 spermatozoa were randomly counted using an optical microscope. Spermatozoa stained blue were considered to be spermatozoa with an immature nucleus.

### Assessment of sperm chromatin structure by toluidine blue staining

Briefly, for each sample, two washed spermatozoa smears, freshly fixed with 96% ethanol-acetone were taken. After 12 h, the slides were treated with 0.1 M HCl at 4 °C for 5 min and washed with distilled water (3 times for 2 min each). The slides were then covered with Toluidine Blue (TB) solution (0.05% TB in 50% McIlvain citrate phosphate buffer, pH 3.5–4) for 5–10 min and washed with distilled water. Dehydration of the slides was carried out in successive ethanol baths (70, 96 and 100%). Finally, the slides were covered and mounted with xylene at room temperature (2–3 min), and the spermatozoa were counted under an optical microscope. For each sample, 200–500 spermatozoa were evaluated. Dark blue stained spermatozoa were considered to have abnormal chromatin packaging [49].

### Statistical analysis

For the statistical analyses, we used the Statistical Package for the Social Sciences (SPSS software, version 22; Chicago, IL, USA). All parameters had a normal distribution. For the descriptive analysis of the results, the data were expressed as mean ± standard deviation (SD). In addition, Pearson analysis was used to present correlations between the different parameters. Differences between means were evaluated using ANOVA (*P-value < 0.05*). To determine which means were statistically different from the others the Student *t-*test for pairwise comparisons was used. *P* values less than 0.05 were considered statistically significant.

## Results

### Experiment 1

**Fig. 1:**
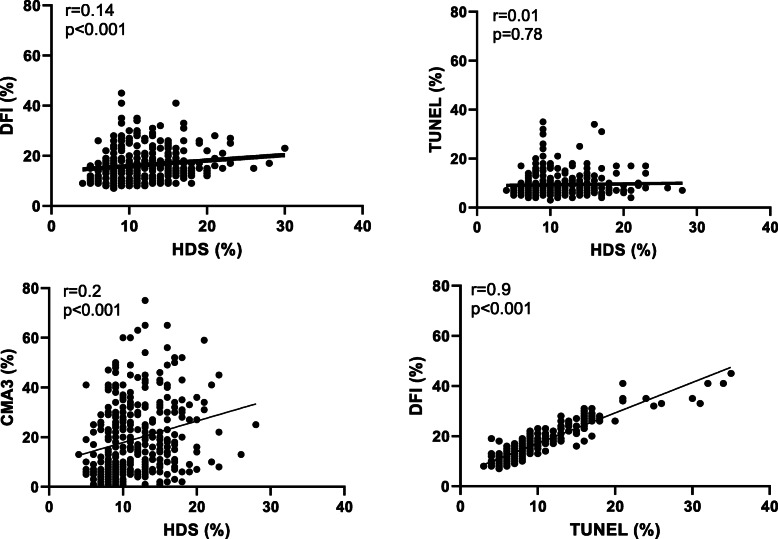
Correlations analysis between TUNEL, DFI, HDS and CMA3 assays. DFI versus HDS (upper left graph). TUNEL versus HDS (upper right graph). CMA3 versus HDS (lower left graph). DFI versus TUNEL (lower right graph). TUNEL: Terminal deoxynucleotidyl transferase dUTP nick end labeling, CMA3: Chromomycin A3; HDS: high DNA stainability; DFI: DNA fragmentation index

Table 1 presents the parameters monitored for the entire cohort, with the exception of 22 samples for which complete data were not available (final cohort size: *N* = 375). For each parameter measured, Table 1 gives the mean (+/− SD) as well as the min and max values within the cohort. Table 2 shows the correlations between semen/sperm parameters (semen volume, sperm concentration, total abnormal morphology [%], abnormal head morphology [%] and total motility [%]) and the criteria assessed (sperm DNA fragmentation as monitored by TUNEL and SCSA®, sperm nuclear condensation as revealed by HDS and protamine deficiency [CMA3]). It appears that “total motility” has low negative correlations with all tests [TUNEL (*r* = − 0.11; *p* = 0.03), DFI (r = − 0.17; *p* < 0.001), HDS (*r* = − 0.12; *p* = 0.01)] and CMA3 (*r* = − 0.11; *p* = 0.02)]. As it could be expected, “abnormal sperm morphology” and “abnormal sperm head morphology” showed positive correlations with HDS [(*r* = 0.16, *p* < 0.001), *r* = 0.17, *p* < 0.001, respectively) but these correlations were also quite weak. It should be noted that in both cases (“abnormal sperm morphology” and “abnormal head morphology”), the correlations were stronger with CMA3 (*r* > 0.2; see Table 2). CMA3 had a mean negative correlation with “sperm concentration” (*r* = − 0.22; *p* < 0.001). A weak positive correlation was also observed between “sperm volume” and “DFI” (*p* < 0.05). In response to the question whether TUNEL, DFI, HDS and CMA3 were correlated with each other, we found (see Fig. 1) that HDS was weakly positively correlated with DFI and CMA3 (*r* = 0.14, *p* < 0.001 and *r* = 0.2, *p* < 0.001, respectively) while it was not correlated with TUNEL at all (*r* = 0.01, *p* = 0.78). On the contrary, TUNEL was highly positively correlated with DFI (*r* = 0.9, *p* < 0.001).

**Table 1:** Global cohort analyses (*N* = 375)

Parameters	***Mean ± SD***	***Minimum***	Maximum
Semen volume [ml]	4.09 ± 1.72	0.7	10.2
Sperm concentration [10^6^/ml]	55.13 ± 37.34	1.0	192
Sperm count [10^6^ /ejaculate]	219.09 ± 170.5	3.08	940.8
Sperm abnormal morphology [%]	96.55 ± 1.58	90	100
Total motility [%]	43.46 ± 19.96	0	96.5
Progressive motility [%]	0.53 ± 1.56	0	9.4
Non-progressive motility [%]	42.89 ± 19.79	0	96.5
Immotile [%]	56.53 ± 20.16	3.5	100
DNA fragmentation (TUNEL) [%]	9.26 ± 4.63	3	35
DNA fragmentation index (DFI) [%]	16.18 ± 6.1	7.0	45.0
High DNA Stainability (HDS) [%]	11.57 ± 3.98	4.0	28
Protamine deficiency (CMA3) [%]	19.64 ± 15.31	1	75

*TUNEL* Terminal deoxynucleotidyl transferase dUTP nick end labeling, *CMA3* Chromomycin A3

**Table 2:** Correlation analyses between sperm DNA integrity tests and conventional semen evaluation parameters

Correlations N = 375	Sperm DNA fragmentation (TUNEL)	DNA fragmentation index (DFI)	High DNA Stainability (HDS)	Protamine deficiency (CMA3)
Semen volume [ml]	0.09	0.11^b^	−0.07	−0.09
Sperm concentration [10^6^/ml]	0.01	−0.04	−0.09	− 0.22^a^
Sperm abnormality morphology [%]	0.02	0.05	0.16^a^	0.27^a^
Sperm abnormal head [%]	0.03	0.06	0.17^a^	0.25^a^
Total motility [%]	−0.11^b^	−0.17^a^	−0.12^b^	− 0.11^b^

*TUNEL* Terminal deoxynucleotidyl transferase dUTP nick end labeling, *CMA3* Chromomycin A3

^a^ Correlation is significant at the 0.01 level (2-tailed)

^b^ Correlation is significant at the 0.05 level (2-tailed)

We also analyzed the DFI, HDS and TUNEL tests after grouping the cohort into age groups (Table 3). The age classes were arbitrarily selected as follows (21/30; 31/40; 41/50 and 51/65). The distribution of patients was so low in the last class (51/65; *N* = 8) that we had to exclude it from the analysis. As we will see later and, as reported elsewhere, only DFI was correlated with age classes and showed that it increased when the cohort (21/30) was compared to the two older cohorts (Table 3). None of the other parameters (HDS and TUNEL) appeared to be significantly correlated with age in this study.

**Table 3:** TUNEL, DFI, HDS and AGE after grouping into age classes

AGE Class	Mean	SD	N
**AGE**			
21➔30	28.20	2.23	51
31➔40	35.62	2.73	214
41➔50	43.67	2.49	79
*51➔65*	*54.62*	*4.41*	*8*
**TUNEL**			
21➔30	8.39	3.85	44
31➔40	9.26	4.52	195
41➔50	9.18	5.06	73
ANOVA *p* = 0.51			
**DFI**			
21➔30	14.67	5.64	49
31➔40	16.25*	6.09	207
41➔50	16.22*	6.02	76
ANOVA *p* = 0.24			
*Student *t*: *p* < 0.05			
**HDS**			
21➔30	11.53	4.07	49
31➔40	11.49	3.91	206
41➔50	11.90	4.32	76
ANOVA *p* = 0.74			

We then decided to examine the behavior of DFI, HDS, TUNEL and CMA3 in relation to the WHO catergorization of seminal samples. As shown in Table 4, we were able to analyze with sufficient confidence the normozoospermic (N), asthenozoospermic (A), teratozoospermic (T) and, astheno-teratozoospermic (AT) subcohorts as they were represented with sufficient numbers of patients (93, 45, 97 and 61, respectively). Table 4 shows that DFI, TUNEL and CMA3 showed significant differences when the subcohorts were compared (ANOVA tests, Table 4) whereas this was not the case with HDS. By pairwise comparison of each subcohort using a bilateral student t-test, we show that DFI and TUNEL are both significantly different when the normozoospermic (N) subcohort was compared to any pathological subcohort (T, A, AT). For the CMA3 test, it was only statistically different when the N subcohort was compared to the teratozoospermic (T) subcohort but not to the A and AT subcohorts.

**Table 4:** Correlation between DFI, HDS, TUNEL and CMA3 assays and clinical infertility situations

	DFI	HDS	TUNEL	CMA3
*p* ANOVA1	*0,003*	*0,058*	*0,012*	*0,002*
test *t* N vs T	0,021	0,087	0,021	0,001
test *t* N vs AT	0,001	0,070	0,001	0,157
test *t* N vs A	0,001	0,402	0,004	0,947

*N* Normozoospermic, *T* Teratozoospermic, *AT* Astheno-teratozoospermic, *A* Asthenozoospermic

### Experiment II

**Fig. 2:**
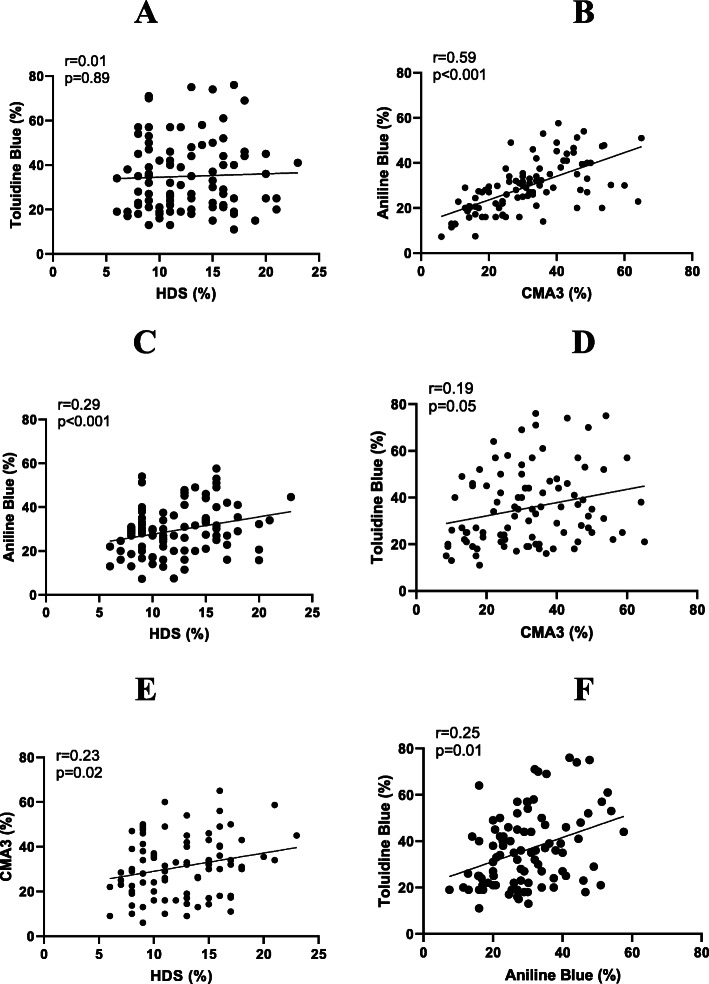
Correlations analysis between HDS and sperm chromatin maturity tests. **a** Toluidine blue versus HDS. **b** Anilin blue versus CMA3. **c** Anilin blue versus HDS. **d** Toluidine blue versus CMA3. **e** CMA3 versus HDS. **f** Toluidine blue versus Anilin blue. HDS: High DNA Stainability; CMA3: Chromomycin A3. TUNEL: Terminal deoxynucleotidyl transferase dUTP nick end labeling

In this study, 100 samples were further analyzed for sperm nuclear compaction by staining with aniline blue (AB) and toluidine blue (TB). Table 5 shows the mean (+/− SD) as well as the min and max values of the cohort for all parameters measured. To check whether CMA3, AB and TB, all of which evaluate sperm nuclear compaction in some way, are correlated, a correlation analysis was performed (see Fig. 2a). AB showed a fairly good correlation with CMA3 (*r* = 0.59, *p* < 0.001) while TB showed a poor correlation (*r* = 0.19, *p* = 0.05). Compared to each other, AB and TB showed a low to medium correlation (*r* = 0.25, *p* = 0.01).

**Table 5:** Sub-cohort analyses (*N* = 100)

Parameters	***Mean ± SD***	***Minimum***	***Maximum***
Semen volume [ml]	4.69 ± 5.52	0.70	40.70
Sperm concentration [10^6^/ml]	53.67 ± 33.85	7.00	156.00
Sperm count [10^6^ /ejaculate]	238.62 ± 291.69	18.00	2604.8
Sperm abnormal morphology [%]	96.42 ± 1.52	90	100
Total motility [%]	46.6 ± 22.16	0.00	91.2
Progressive motility [%]	0.47 ± 1.45	0.00	7.7
Non-progressive motility [%]	46.39 ± 22.16	0.00	90.7
Immotile [%]	52.93 ± 22.48	8.80	100
Sperm DNA fragmentation (TUNEL) [%]	9.54 ± 5.01	4.00	34
DNA fragmentation index (DFI) [%]	17.15 ± 5.52	10.00	41.00
High DNA Stainability (HDS) [%]	12.51 ± 3.9	6.00	23
Protamine deficiency (CMA3) [%]	30.82 ± 13.92	6.00	65
Aniline blue [%]	29.69 ± 11.37	7.30	57.60
Toluidine blue [%]	35.72 ± 16.46	11.00	76.00

*SD* Standard deviation, *TUNEL* Terminal deoxynucleotidyl transferase dUTP nick end labeling

In examining the correlations between these three tests and the sperm parameters, we found that all three tests showed the same types of moderately positive correlations with “abnormal sperm morphology” and “abnormal sperm head morphology” (Table 6). All three tests also showed a low (TB) to medium (AB and CMA3) negative correlation with “sperm concentration” (Table 6). The strongest correlations were observed with AB. Finally, only CMA3 showed a weak negative correlation with “total motility”.

**Table 6:** Correlation analyses between sperm nuclear condensation/compaction tests and conventional semen evaluation parameters

Parameters (***N*** = 100)	AB (%)	TB (%)	CMA3 (%)
Semen volume (ml)	0.02	−0.01	0.06
Sperm concentration (10^6^/ml)	−0.36^a^	−0.1	−0.3^a^
Sperm abnormal morphology (%)	0.36^a^	0.30^a^	0.23^b^
Sperm abnormal head morphology (%)	0.36^a^	0.28^a^	0.23^b^
Total motility (%)	−0.13	0.00	−0.20^b^

*AB* Aniline blue, *TB* Toluidine blue, *CMA3* Chromomycine A3

^a^ Correlation is significant at the 0.01 level (2-tailed)

^b^ Correlation is significant at the 0.05 level (2-tailed)

When asked whether these three tests (AB, TB, and CMA3) correlated with the HDS parameter from the SCSA® analysis, Fig. 2b shows that TB was not correlated at all with HDS, whereas both AB and CMA3 showed weak to moderate correlations with HDS (*r* = 0.29, *p* < 0.001 and *r* = 0.23, < 0.02, respectively). AB is the assay that showed the highest correlation with HDS.

## Discussion

From the data we report here, we observed that HDS was weakly correlated with the percentages of spermatozoa with “abnormal morphology” or, in a smaller fraction, “abnormal head morphology”. This does not support the idea that HDS reflects the immaturity of the sperm nucleus due to a non-optimal histone to protamine ratio. If this were the case, sperm nuclei with a lower amount of protamine should be less condensed and therefore have abnormal head morphology. However, it could be argued that the change in histone to protamine ratio encountered in AO-sensitive HDS sperm is not sufficient to significantly alter the compaction of the sperm nuclei such that they appear with abnormal head morphology. We also found that in the two cohorts studied (*N* = 375 and *N* = 100), HDS was weakly correlated with CMA3. CMA3 directly competes with protamines in the sperm nucleus to bind GC-rich DNA domains, giving an indirect indication of protamine deficiency [50]. In addition, we show that HDS does not correlate at all with toluidine blue (TB) staining although some authors have reported the opposite [51]. The TB stain is thought to allow the identification of abnormally packed and low-density chromatin due to the greater accessibility of TB to the phosphate groups of DNA [52]. In addition, we also found that HDS did not correlate well with AB staining. Unlike TB, the AB stain gives an indication of the nuclear histone content of the semen. As histones contain a high number of lysine residues, they confer alkaline properties that allow interaction with acidic AB. Sperm with a high level of residual histones (to the detriment of protamine) are therefore reactive to AB. In contrast, we found that AB and CMA3 correlated rather well (*r* = 0.59, *p* < 0.001) as reported elsewhere [53], whereas TB correlated poorly with CMA3 and AB. This is perhaps not too surprising since AB and CMA3 both address the nuclear proteins histone and protamine, respectively, whereas TB is an indirect indicator of nuclear compaction that is not related to nuclear protein occupation and could therefore be influenced by other factors. In our analysis, CMA3 was found to be significantly higher in the teratozoospermic (T) subcohort when compared with the normozoospermic (N) subcohort (Table 4). This is logical because much of teratozoospermia is represented by abnormal sperm head morphology. The HDS was not found to be different when the N subcohort was compared to the T subcohort (Table 4), suggesting that the HDS does not discriminate well against spermatozoa with abnormal head morphology. Overall, the lack of strong correlations between HDS, abnormal sperm head morphology, CMA3, AB and TB staining does not allow this parameter to be used with confidence as a strong predictor of sperm nuclear integrity.

Interestingly, HDS was found to be weakly correlated with DFI in the cohorts studied. In addition, HDS was not correlated at all with TUNEL, while DFI and TUNEL were strongly correlated. The strong correlation found between DFI and TUNEL is not surprising, as these two tests have already repeatedly shown a fairly good correlation, although they do not address the issue of sperm DNA integrity in the same way (e.g. see: [54]). The low correlation of TUNEL and DFI with the HDS is a bit more confusing. Based on the literature, it is commonly accepted that if there are more DNA breaks, the level of condensation of the sperm nucleus will be lower, especially under slightly denaturing conditions such as in the SCSA® test [55–57]. If this had been the case, the DFI, the TUNEL and the HDS should be correlated and would have behave in the same way, meaning that when the TUNEL or DFI increases, the HDS should increase. It is not the case. Consistent with our observations, others have reported that there is no correlation between HDS, TUNEL, DFI, CMA3, AB and TB [58, 59]. However, some positive correlations have also been reported [60–62]. It should be noted, however, that these positive correlations were again very low and may be explained by various factors such as: sample size, specific sub-populations studied, procedure used for evaluation (microscopy vs. flow cytometry).

The absence or weak correlations between tests assessing the level of sperm DNA fragmentation and tests assessing sperm nuclear condensation such as HDS, AB and CMA3 suggest that sperm DNA fragmentation is not strongly associated with optimal sperm nuclear condensation. This is perfectly possible because optimal sperm nucleus condensation is ensured by several distinct but nevertheless partially interconnected mechanisms. One of these is the protamine content, which allows for a large reduction in the size of the sperm nucleus. The second mechanism is the extensive disulfide bridging that occurs during epididymal maturation of the spermatozoa, which links protamine rings and locks-up the sperm nucleus in a condensed state [13]. The availability of zinc also contributes to this optimal condensation of the sperm nucleus by preserving some free protamine thiols from oxidation into disulfide bridges [63]. In this picture, disulfide bridges and thiol groups interacting with zinc both contribute to define an optimal level of sperm nuclear condensation [64]. It has been shown that situations that would facilitate extraction of sperm chromatin zinc (such has long exposure to the zinc chelating seminal vesicular fluid as it could happened commonly in ART clinics) may alter this equilibrium [64]. The role played by zinc in sperm nuclear condensation could be considered as a limitation in our study since we did not evaluate in any sample the chromatin zinc content or the degree of zinc depletion in the semen. To some extent it may explain the observed inter- and intra-study variations [65]. The third is the extent of DNA breaks that can affect this complex organization. Thus, the nuclear immaturity of spermatozoa or its susceptibility to denaturing conditions may have different origins that cannot be assessed by a single test. For example, the oxidative stress so often associated with male infertility [66, 67] may partly explain these weak or absent correlations. Under moderate oxidative stress, it is perfectly possible to increase sperm DNA/nuclear condensation, whereas under high oxidative stress, a decrease in sperm DNA/nuclear compaction is observed. This has been clearly demonstrated in animal models [68–70]. In addition to the above possibilities, another confounding factor that could affect the results is the length of abstinence. It is known that older sperm have greater DNA fragmentation and repeated ejaculations may reduce the level of sperm nucleus fragmentation. Given that all studies attempt to obtain sperm with an abstinence time according to the WHO recommendation, as we have done here, we remain consistent here with the literature. It is clear that further studies will be needed to see if the relationships observed are applicable to semen samples outside this range of abstinence time and in particular less “old”.

It should be noted that a recent report in which the SCSA® test was used on a rather large cohort of 6881 men classified by age group came to an interesting conclusion [71]. The authors reported that, as expected and as shown elsewhere with smaller cohorts, DFI increased with age but HDS decreased with age. A similar decrease in HDS with age was also reported for a very large cohort of SCSA® data with more than 25,000 entries (American Society for Reproductive Medicine = ASRM 2018 communication [72]). This situation of increasing DFI and decreasing HDS with age indicates that despite the increase in DNA breaks, the sperm nuclei of aging men are more condensed, resulting in lower HDS. It is unlikely that this is due to a higher incorporation of protamine into the sperm nucleus of aging men, although this needs to be verified. More likely, it could be related to a redox problem, as it is well known that oxidative stress increases with age. According to the free radical theory of aging, age-related physiological changes including an increase in general inflammatory status and deficiencies in the systems in charge of elimination of reactive oxygen species (ROS) from mitochondrial metabolism expose the entire body, including the reproductive system, to the deleterious effects of ROS [73]. In this regard, Paoli et al. suggested that ROS are at the root of replication errors during cycles of spermatogenetic divisions that increase with age [74]. A decrease in the transcriptional efficiency of protamines in spermiognenesis as well as post-transcriptional impacts via the modification of small non-coding RNA profiles contained in spermatozoa over age (as recently published in an animal model [75]) may be the source of a certain nuclear heterogeneity resulting in abnormalities in chromatin condensation [76]. Separating our cohort (*N* = 375) by age, we also found that DFI increased significantly between the 21–30 age group compared to the two older groups, 31–40 and 41–50 (see Table 3). TUNEL and HDS were not statistically different in the three age groups studied, which in our case could be attributed to the small size of the cohort and its narrow distribution.

## Conclusions

Overall, our observations point out that HDS is not a reliable indicator of defective sperm nuclear compaction (i.e., reflecting nuclear maturity) and, as may be assumed too quickly, of protamine deficiency or abnormally high histone levels. If this had been the case, one would have expected a strong correlation of HDS with CMA3 and AB staining. None of the correlations we measured between HDS, AB and CMA3 (Fig. 2) were greater than 0.3, which is rather low. This potentially reflects the fact that HDS may be related to sperm nuclear instability which is not solely due to its nuclear protein content. Some authors believe that HDS may be of interest and have reported that individuals with an HDS greater than 25% have a higher risk of miscarriage and poor live birth outcomes [77], others found that this was only valid in ICSI but not in conventional IVF cycles [78]. However, the reported correlations were again quite weak as the authors themselves pointed out [78], which, in our view, supports the idea that the clinical relevance of HDS is weak.

## Data Availability

All data generated or analyzed in this study are provided.

## Abbreviations

AB: Aniline blue
AO: Acridine orange
ART: Assisted reproductive technology
ASRM: American society for reproductive medicine
CMA3: Chromomycin A3
DFI: DNA fragmentation index
DNA: Deoxyribo nucleic acid
dUTP: Deoxyuridine triphosphate
EDTA: Ethylene diamine tetra-acetate
HCl: Hydrochloric acid
HDS: High DNA stainability
ICSI: Intracytoplasmic sperm injection
IUI: Intrauterine insemination
IVF: In vitro fertilization
OAT: Oligoasthenoteratozoospermia
PBS: Phosphate buffer saline
RNA: Ribonucleic acid
SCSA: Sperm chromatin structure assay
TB: Toluidine blue
TESE: Testicular sperm extraction
TNE: Tris NaCl EDTA
TUNEL: Terminal deoxynucleotidyl transferase-mediated deoxyuridine triphosphate-biotin nick end labeling
WHO: World health organization
